# Evaluation of Muscle Mass and Stiffness with Limb Ultrasound in COVID-19 Survivors

**DOI:** 10.3389/fendo.2022.801133

**Published:** 2022-02-17

**Authors:** Sarah Damanti, Marta Cilla, Bruno Tuscano, Rebecca De Lorenzo, Giuseppina Manganaro, Aurora Merolla, Giacomo Pacioni, Chiara Pomaranzi, Valeria Tiraferri, Sabina Martinenghi, Giordano Vitali, Emanuele Bosi, Caterina Conte, Andrea Giustina, Moreno Tresoldi, Patrizia Rovere Querini

**Affiliations:** ^1^ Unit of General Medicine and Advanced Care, Instituto di Ricovero e Cura a Carattere Scientifico (IRCSS) San Raffaele Institute, Milan, Italy; ^2^ Unit of Radiology, IRCCS San Raffaele Institute, Milan, Italy; ^3^ Vita-Salute San Raffaele University, Milan, Italy; ^4^ San Raffaele Diabetes Research Institute, Instituto di Ricovero e Cura a Carattere Scientifico (IRCSS) Ospedale San Raffaele, Milan, Italy; ^5^ Department of Human Sciences and Promotion of the Quality of Life, San Raffaele Roma Open University, Rome, Italy; ^6^ Department of Endocrinology, Nutrition and Metabolic Diseases, Instituto di Ricovero e Cura a Carattere Scientifico (IRCSS) MultiMedica, Milan, Italy; ^7^ Institute of Endocrine and Metabolic Sciences, San Raffaele Vita-Salute University, Instituto di Ricovero e Cura a Carattere Scientifico (IRCSS) San Raffaele Hospital, Ospedale San Raffaele, Milan, Italy; ^8^ Department of Immunology, Transplantation and Infectious Diseases, Instituto di Ricovero e Cura a Carattere Scientifico (IRCSS) Ospedale San Raffaele, Milan, Italy

**Keywords:** muscle, ultrasound, sarcopenia, COVID - 19, muscle mass, muscle quality

## Abstract

**Background:**

acute illnesses, like COVID-19, can act as a catabolic stimulus on muscles. So far, no study has evaluated muscle mass and quality through limb ultrasound in post-COVID-19 patients.

**Methods:**

cross sectional observational study, including patients seen one month after hospital discharge for SARS-CoV-2 pneumonia. The patients underwent a multidimensional evaluation. Moreover, we performed dominant medial gastrocnemius ultrasound (US) to characterize their muscle mass and quality.

**Results:**

two hundred fifty-nine individuals (median age 67, 59.8% males) were included in the study. COVID-19 survivors with reduced muscle strength had a lower muscle US thickness (1.6 versus 1.73 cm, p =0.02) and a higher muscle stiffness (87 versus 76.3, p = 0.004) compared to patients with normal muscle strength. Also, patients with reduced Short Physical Performance Battery (SPPB) scores had a lower muscle US thickness (1.3 versus 1.71 cm, p = 0.01) and a higher muscle stiffness (104.9 versus 81.07, p = 0.04) compared to individuals with normal SPPB scores. The finding of increased muscle stiffness was also confirmed in patients with a pathological value (≥ 4) at the sarcopenia screening tool SARC-F (103.0 versus 79.55, p < 0.001). Muscle stiffness emerged as a significant predictor of probable sarcopenia (adjusted OR 1.02, 95% C.I. 1.002 – 1.04, p = 0.03). The optimal ultrasound cut-offs for probable sarcopenia were 1.51 cm for muscle thickness (p= 0.017) and 73.95 for muscle stiffness (p = 0.004).

**Discussion:**

we described muscle ultrasound characteristics in post COVID-19 patients. Muscle ultrasound could be an innovative tool to assess muscle mass and quality in this population. Our preliminary findings need to be confirmed by future studies comparing muscle ultrasound with already validated techniques for measuring muscle mass and quality.

## Background

Sarcopenia is a progressive and generalized skeletal muscle disorder, characterized by an accelerated loss of muscle mass and function, which is associated with an increased likelihood of developing adverse outcomes (1–6). Decline in muscle mass is not homogenous across different body anatomic regions, as sarcopenia occurs earlier in the lower limbs (7). Moreover, muscle quality, which is precociously impaired in sarcopenia (2), has an important impact on muscle function (8) and clinical outcomes (9), independently from muscle mass reduction. Muscle quality is determined by micro- and macroscopic changes in muscle architecture and composition (8, 10). In research settings, magnetic resonance imaging (MRI) and computed tomography (CT) have been used to study muscle quality, by assessing fat infiltration into muscle, and evaluating muscle attenuation (11, 12). On the other hand, muscle quality has also been defined in functional terms, as the muscle strength delivered per unit of muscle mass (13, 14) or volume (15). Because of its effects on muscle performance and clinical outcomes, muscle quality should always be considered in the assessment of sarcopenic subjects. Unfortunately, there has not been universal consensus yet on which method should be used for the evaluation of muscle quality in the routine clinical practice (1).

Acute illnesses, like COVID-19, can act as a catabolic stimulus on muscles (16, 17). Indeed, weight loss has been reported to be pronounced in COVID-19 patients (17, 18) who are at high risk of developing acute sarcopenia (19, 20). However, the degree of muscle mass and functional loss depends on multiple factors: preexisting conditions (*i.e.* age, frailty, comorbidities), the degree of inflammatory response to the SARS-CoV-2 infection, anorexia, inadequate protein supply, and physical inactivity during the active phase of the COVID-19 disease (21). Old, comorbid and frail patients are at higher risk of developing acute sarcopenia, even in the presence of a mild COVID-19 disease. Anyway, acute sarcopenia can occur in previously robust individuals too (16, 22, 23).

Acute sarcopenia augments patients’ vulnerability to stressors (24, 25), increasing their risk of developing adverse outcomes. Moreover, acute sarcopenia can evolve into chronic sarcopenia (26), a condition closely related to frailty (24).

Skeletal muscle ultrasound is an accurate imaging technique (27) for evaluating muscle architecture, and for quantifying muscle mass, as demonstrated by its comparison with direct anatomical assessment on cadavers (28, 29) and MRI studies (30). However, its role in diagnosing sarcopenia is just speculative. None of the current definitions of sarcopenia includes muscle echography in its diagnostic algorithms (1, 31, 32), and the analysis of current medical literature highlights the absence of a standardized method for performing muscle ultrasonography, to detect sarcopenia in clinical practice (33). In addition, normative values for defining lower limb ultrasound quantity and quality are lacking.

Finally, no study has evaluated muscle mass in post COVID-19 patients, through limb ultrasound, so far. The main objective of our study was evaluating muscle mass and quality through lower limb ultrasound in a cohort of COVID-19 survivors. As secondary objectives we performed i) a correlation of the muscle ultrasound parameters with validated measures of muscle function, nutritional status and inflammatory indexes during hospital stay, ii) an assessment of the association between muscle ultrasound parameters and probable sarcopenia and iii) a definition of ultrasound parameters cut-off values associated with probable sarcopenia.

## Material and Methods

This was a cross sectional observational study. We evaluated patients attending a dedicated post-COVID-19 outpatient clinic, who were previously hospitalized for SARS-CoV-2 pneumonia in the Internal Medicine Departments of the San Raffaele University Hospital, Milan, Italy (34). The data presented in this study were collected in the visits that took place one month after hospital discharges, from the 15^th^ April 2021 till the 15^th^ July 2021. We are still collecting data on three- and six-months follow-ups. The present study was part of the COVID-BioB study (NCT04318366), which aimed at characterizing hospitalized COVID-19 patients, through the prospective collection of several demographic, anthropometric, clinical and laboratory variables, as previously described (35). The COVID-BioB study protocol was approved by the San Raffaele University Hospital Ethics Committee (protocol no. 34/int/2020). A convenience sample size was used due to the setting of the COVID-19 pandemic.

During the follow-up visits, the patients underwent a multidimensional evaluation, consisting in: medical history, including self-reported weight loss during hospitalization, physical examination, anthropometric measurements to calculate the body mass index (BMI), before hospital stay, and one month after hospital discharge, screening for sarcopenia through the Strength, Assistance with walking, Rising from a chair, Climbing stairs, and Falls (SARC-F) questionnaire (36), assessment of muscle strength through the hand grip strength test (37), evaluation of muscle performance with the Short Physical Performance Battery (SPPB) test (38) and screening for malnutrition with the Mini Nutritional Assessment Short Form (MNA-SF) questionnaire (39). Patients suffering from dementia were generally helped by their care-givers in the compilation of the questionnaires.

Finally, all patients underwent muscle ultrasound of the dominant medial gastrocnemius, to assess muscle mass and quality. We chose to evaluate gastrocnemius muscle, because it has a pennate architecture, thus allowing the assessment of pennation angle. Pennation angle is the angle formed at the fiber insertions into deep aponeurosis in pennate muscles and it is strongly correlated to muscle mass. Pennation angle was automatically calculated by the ultrasound software after the sonographer had manually identified the angle formed between muscle fiber insertions and deep aponeurosis.

By limb ultrasound, muscle quality can be assessed either with the determination of muscle echogenicity (40, 41), or with muscle stiffness (42). However, there is no consensus on which of these parameters should be preferred, in the assessment of muscle quality by limb ultrasound. Therefore, since our radiologists had more experience in the evaluation of muscle stiffness, we chose to investigate this aspect of muscle quality.

During muscle ultrasound, the patients laid prone on the examination couch, with the foot positioned perpendicularly to the tibia outside the couch. The ultrasound examinations were performed by two trained sonographers (SD and MC). To improve acoustic coupling, abundant water-soluble transmission gel was used on the linear array probe (7-10 MHz, General Electric model), using B-mode. The probe was set perpendicularly to the dermal surface, to get images, including both superficial and deep aponeurosis, and with an orientation coinciding with that of the muscle fascicles between the aponeuroses. Images were obtained along the mid-sagittal line of the medial gastrocnemius at the mid-distance between its proximal and distal tendon insertions (43, 44). Depth was initially set at 30 mm, then it was modified during the examination (range: 30-60 mm) to visualize the entire muscle thickness. Resting Euclidean distance between the internal borders of the superficial and deep aponeuroses (i.e. muscle thickness) was assessed in three points of the muscle, equally spaced along the image, and a mean value was calculated. In addition, the angle between the fascicle and the deep aponeurosis (i.e. pennation angle) was calculated.

Images were stored as DICOM files, and transferred to a computer for processing. Muscle stiffness was measured by means of an AGFA Enterprise Imaging program. The size of regions of interest (ROI), to estimate the stiffness index (SI), was set between 0.2 and 0.3 cm². ROI were measured in three points of the medial gastrocnemius, equally spaced along the images. Then a mean value of the three SI obtained, was calculated.

### Statistical Analyses

The baseline characteristics of the study population, the main aspects of the COVID-19 hospitalization, muscle and nutritional parameters one month after hospital discharge were described through descriptive statistics. Continuous variables were presented as mean and standard deviation (SD), when normally distributed, or with median and interquartile range (IQR), when data had a skewed distribution. Dichotomous variables were presented as number (N) and percentage (%). In addition, we performed a comparison of the distribution of categorical and continuous variables among patients with reduced (SPPB ≤8) versus normal (SPPB >8) muscle performance, reduced (Hand Grip Strength < 27 kg in men or < 16 kg in women) versus normal (Hand Grip Strength ≥ 27 kg in men or ≥ 16 kg in women) muscle strength and pathological (SARC-F ≥ 4) or normal (SARC-F < 4) values of the sarcopenia screening tool. Comparisons were made with the chi-squared test for categorical variables, and with the Mann-Whitney U test, for continuous variables. Cut-off values for muscle performance and strength were chosen, according to the European Working Group on Sarcopenia Guidelines (1) and to literature data (36).

We assessed the correlations between muscle ultrasound characteristics (muscle thickness, pennation angle and muscle stiffness), measures of muscle function (SPPB and Hand Grip Strength), nutritional status (MNA-SF), age, SARC-F, inflammatory indexes [highest C Reactive Protein (CRP) and number of days with CRP above the upper normal limit, highest ferritin during hospital stay, highest white blood cells during hospital stay (WBC)] and length of hospital stay through Spearman correlations.

Probable sarcopenia was defined as a reduced muscle strength at the hand grip test, accordingly to the European Working Group on Sarcopenia Guidelines (1). Binary logistic regression analyses were used, to assess the association between muscle ultrasound parameters, and probable sarcopenia. Unadjusted and stepwise adjusted models were performed. Collinearity tests were run before performing the adjusted model; collinear variables were excluded from the multivariable model, and just one proxy of the severity of SARS-CoV-2 infection (Non-Invasive Mechanical Ventilation) and of clinical complexity before hospital admission (number of chronic therapies) were inserted in the model, in addition to the significant predictors at the univariable analyses.

Finally, we performed ROC analyses, to identify optimal cut-off values of muscle ultrasound characteristics (thickness, stiffness and pennation angle) associated with probable sarcopenia. Reduced muscle strength (defined as a Hand Grip Strength < 27 kg in men or < 16 kg in women) was used as state variable. The Area under the curve (AUC) was calculated as a summary of diagnostic accuracy. Maximum value of the Youden’s index was used for selecting the optimum cut-off points.

All statistical analyses were performed with SPSS version 25.0 (SPSS Inc., Chicago, IL, USA).

## Results

Two hundred and fifty-nine patients seen at a dedicated post-COVID-19 outpatient clinic, were included in the study. **Table 1** shows the main baseline characteristics of the study population. **Table 2** illustrates the main characteristics of their COVID-19 hospitalization. **Table 3** provides information on patients’ muscle and nutritional characteristics, one month after hospital discharge.

**Table 1:** Baseline characteristics of the study population and their comparison among groups with pathologic and normal values of a sarcopenia screening tool,muscle strength and performance.

	Total sample	SARC-F		p	GRIP STRENGTH		p	SPPB		p
	(N = 259)	≥ 4 (N = 32)	< 4 (N =222)		Low * (N = 121)	Normal (N = 134)		≤ 8 (N = 14)	> 8 (N = 238)	
Age	67 (IQR 56 – 75)	74.5 (IQR 62.5 – 80)	66 (IQR 55.75 – 74)	**0.01**	73 (IQR 63 – 79)	61.0 (IQR 52.0 – 69.0)	**< 0.001**	80.5 (IQR 74 – 83.75)	66.0 (IQR 55.0 – 73.0)	**< 0.001**
Males	155 (59.8%)	5 (15.6%)	147 (66.2%)	**< 0.001**	68 (56.2%)	84 (62.7%)	0.3	5 (35.7%)	148 (62.2%)	**0.049**
Smoke										
*Never*	135 (52.1%)	19 (59.4%)	112 (50.7%)	0.47	61 (50.4%)	72 (53.7%)	0.21	7 (50%)	124 (52%)	0.88
*Previous*	97 (37.5%)	9 (28.1%)	87 (39.2%)		51 (42.1%)	44 (32.8%)		5 (35.7%)	89 (37.4%)	
*Active*	26 (10%)	4 (12.5%)	22 (9.9%)		9 (7.4%)	17 (12.7%)		2 (14.3%)	24 (10.1%)	
Alcohol abuse	6 (2.3%)	0 (0%)	6 (2.7%)	**0.02**	5 (4.1%)	1 (0.7%)	0.11	0 (0%)	6 (2.5%)	0.81
Bedridden before hospital admission	2 (0.8%)	1 (3.1%)	1 (0.5%)	0.11	2 (1.7%)	0 (0%)	0.13	0 (0%)	1 (0.4%)	0.81
Weight (kg) before hospital admission	79 (IQR 69 – 91)	70 (IQR 62.25 – 80.75)	81.0 (IQR 70 – 92)	**0.005**	75 (IQR 65 – 90)	83.0 (SD ± 15.65)	**0.008**	70 (IQR 66.75 – 77.75)	80.0 (IQR 69.5 – 92.0)	**0.04**
BMI (kg/m2) before hospital admission	28 (IQR 24.87 – 31.01)	28 (IQR 23.94 – 31.54)	27.7 (IQR 24.87 – 30.82)	0.81	27.3 (IQR 24.56 – 31.83)	28.1 (IQR 25.12 – 30.32)	0.63	27.1 (IQR 23.65 – 29.38)	28 (IQR 24.99 – 31.14)	0.29
Chronic therapies at hospital admission	3 (IQR 1 – 5)	5.5 (IQR 3.25 – 7.75)	2 (IQR 1 – 5)	**< 0.001**	4.53 (SD ± 3.46)	2 (IQR 0 – 4)	**< 0.001**	6 (IQR 1.75 – 9)	3 (IQR 1 – 5)	**0.02**
Chronic steroid use	10 (3.9%)	3 (9.4%)	7 (3.2%)	0.07	4 (3.3%)	6 (4.5%)	0.67	0 (0%)	10 (4.2%)	0.43
Hypertension	132 (51%)	17 (53.1%)	112 (50.5%)	0.78	70 (57.9%)	59 (44%)	**0.04**	11 (78.6%)	116 (48.7%)	**0.03**
Diabetes	53 (20.5%)	7 (21.9%)	45 (20.3%)	0.83	33 (27.3%)	19 (14.2%)	**0.008**	5 (35.7%)	46 (19.3%)	0.14
CKD	20 (7.7%)	3 (9.7%)	16 (7.3%)	0.68	13 (11%)	6 (4.4%)	0.10	3 (21.4%)	16 (6.8%)	0.12
Arrhythmia	30 (11.6%)	5 (15.6%)	24 (10.8%)	0.42	18 (14.9%)	11 (8.2%)	0.09	5 (35.7%)	23 (9.7%)	**0.003**
Ischemic heart disease	28 (10.8%)	3 (9.4%)	25 (11.3%)	0.75	18 (14.9%)	10 (7.5%)	0.05	4 (28.6%)	24 (10.1%)	**0.03**
Stroke/TIA	12 (4.6%)	3 (9.4%)	9 (4.1%)	0.18	9 (7.4%)	3 (2.2%)	**0.047**	2 (14.3%)	10 (4.2%)	0.08
Peripheral vascular disease	28 (10.8%)	3 (9.4%)	25 (11.3%)	0.75	16 (13.2%)	12 (9%)	0.26	1 (7.1%)	27 (11.3%)	0.63
COPD	15 (5.8%)	2 (6.3%)	13 (5.9%)	0.93	11 (9.1%)	4 (3%)	**0.04**	3 (21.4%)	12 (5%)	**0.01**
Asthma	9 (3.5%)	3 (9.4%)	6 (2.7%)	0.05	4 (3.3%)	5 (3.7%)	0.87	0 (0%)	9 (3.8%)	0.46
Other respiratory disease	17 (6.6%)	2 (6.3%)	14 (6.3%)	0.98	9 (7.4%)	7 (5.2%)	0.44	1 (7.1%)	15 (6.3%)	0.90
Chronic anemia	9 (3.5%)	3 (9.4%)	6 (2.7%)	0.05	6 (5%)	3 (2.2%)	0.23	2 (14.3%)	7 (2.9%)	**0.03**
Osteoporosis	12 (4.6%)	5 (15.6%)	5 (2.3%)	**< 0.001**	8 (6.6%)	4 (3%)	0.16	1 (7.1%)	9 (3.8%)	0.53
Arthrosis	17 (6.6%)	3 (9.4%)	13 (5.9%)	0.44	9 (7.4%)	8 (6%)	0.62	1 (7.1%)	14 (5.9%)	0.85
Rheumatic disease	11 (4.2%)	3 (9.4%)	8 (3.6%)	0.13	5 (4.1%)	6 (4.5%)	0.91	1 (7.1%)	10 (4.2%)	0.60
Dementia	3 (1.2%)	2 (6.3%)	1 (0.5%)	**0.005**	2 (1.7%)	1 (0.7%)	0.49	1 (7.1%)	1 (0.4%)	**0.006**
Other neurologic diseases	22 (8.5%)	5 (15.6%)	17 (7.7%)	0.13	16 (13.2%)	4 (3%)	**0.01**	4 (28.6%)	15 (6.3%)	**0.002**
Psychiatric disease	32 (12.4%)	7 (21.9%)	24 (10.8%)	0.07	20 (16.5%)	12 (9%)	0.06	4 (28.6%)	26 (10.9%)	**0.048**
Vitamin D deficit	15 (5.8%)	5 (15.6%)	9 (4.1%)	**0.008**	11 (9.1%)	4 (3%)	**0.03**	1 (7.1%)	12 (5%)	0.73
Active neoplasm	18 (6.9%)	2 (6.3%)	16 (7.2%)	0.84	10 (8.3%)	8 (6%)	0.45	2 (14.3%)	16 (6.7%)	0.29

*Hand grip strength < 27 kg in men; < 16 kg in woman.

SARC-F,Screening tool for sarcopenia; SPPB, Short Physical Performance Battery; IQR, Inter Quartile Range; BMI, Body Mass Index; CKD, Chronic Kidney Disease; TIA, Transient Ischemic attack; COPD, Chronic Obstructive Pulmonary Disease. Bold = statistically significant (p < 0.05).

**Table 2:** Main characteristics of the COVID-19 hospitalization of the study population and their comparison among groups with pathologic and normal values of a sarcopenia screening tool, muscle strength and performance.

	Total sample	SARC-F		p	GRIP STRENGTH		p	SPPB		p
	(N = 259)	≥ 4 (N = 32)	< 4 (N =222)		Reduced * (N = 121)	Normal (N = 134)		≤ 8 (N = 14)	> 8 (N = 238)	
Weight loss during hospital stay (kg)	5 (IQR 3 - 7.1)	4.25 (IQR 2 - 5)	5.0 (IQR 3.0 - 8.0)	0.22	5 (IQR 3 - 9)	5.0 (IQR 2.0 - 7.0)	0.2	4.5 (IQR 2.75 - 5)	5 (IQR 3.0 - 7.65)	0.56
Length of hospital stay (days)	14 (IQR 9 - 21)	18.5 (IQR 9 – 31.25)	13.0 (IQR 9 - 20.25)	0.19	16 (IQR 10 - 28.5)	11.0 (IQR 8.0 - 17.0)	**< 0.001**	18.5 (IQR 9.75 - 35.25)	13.5 (IQR 9 - 21)	0.13
ICU stay	22 (8.5%)	3 (9.4%)	19 (8.6%)	0.88	15 (12.4%)	7 (5.2%)	**0.04**	3 (21.4%)	19 (8%)	0.08
Length of ICU stay	14 (IQR 6.5 – 29.5)	18 (IQR 18 - 18)	10.0 (IQR 5.75 - 32.50)	0.76	17 (IQR 8 -33)	9.0 (IQR 5.0 - 19.0)	0.08	17 (IQR 17 - 17)	11.0 (IQR 6.0 - 32.0)	0.96
NIV	67 (25.9%)	7 (21.9%)	59 (26.6%)	0.57	40 (33.1%)	26 (19.4%)	**0.01**	2 (14.3%)	64 (26.9%)	0.30
Steroid use during hospital stay	242 (93.5%)	29 (90.6%)	208 (93.7%)	0.52	113 (93.4%)	125 (93.3%)	0.99	14 (100%)	222 (93.3%)	0.32
Anakinra use	36 (13.9%)	1 (3.1%)	33 (14.9%)	0.07	16 (13.2%)	18 (13.4%)	0.86	1 (7.1%)	34 (14.3%)	0.45
Highest CRP during hospital stay (mg/l)	70.7 (IQR 40.5 - 119.2)	68.3 (IQR 42.0 - 125.87)	68.5 (IQR 39.95 - 118.85)	0.70	81.1 (IQR 40.75 - 131.35)	63.0 (IQR 37.52 - 103.35)	0.06	81.25 (IQR 55.10 - 169.65)	69.8 (IQR 40.67 - 117.52)	0.30
Days with CRP above the normal limit	8 (IQR 6 - 13)	10 (IQR 6.0 - 19.5)	8.0 (IQR 6 - 12)	0.13	10 (IQR 6 - 20)	8.0 (IQR 5.0 - 10.0)	**< 0.001**	11 (IQR 7 - 25.75)	8.0 (IQR 6.0 - 12.75)	**0.03**
Highest ferritin during hospital stay (ng/ml)	943 (IQR 516 - 1522)	639 (IQR 291.0 - 1312.0)	968.0 (IQR 592.0 - 1538.0)	**0.03**	934 (IQR 488.75 - 1654.0)	919.0 (IQR 520.0 - 1370.0)	0.50	556 (IQR 217 - 2096)	960.0 (IQR 549.0 - 1522.0)	0.31
Highest WBC during hospital stay (10^3cells/mmc)	9.9 (IQR 8.0 - 12.9)	9.3 (IQR 7.55 - 12.80)	10.2 (IQR 8.1 - 12.9)	0.57	10.7 (IQR 8.75 - 13.25)	9.5 (IQR 7.4 - 12.4)	**0.01**	9.35 (IQR 8.77 - 12.65)	10.1 (IQR 8.0 - 12.9)	0.88

*Hand grip strength < 27 kg in men; < 16 kg in woman.

SARC-F,Screening tool for sarcopenia; SPPB, Short Physical Performance Battery; IQR, Inter Quartile Range; ICU, Intensive Care Unit; NIV, Non Invasive Ventilation; CRP, C Reactive Protein; WBC, White Blood Cells. Bold = statistically significant (p < 0.05).

**Table 3:** Muscle and nutritional characteristics one month after hospital discharge of the study population and their comparison among groups with pathologic and normal values of a sarcopenia screening tool, muscle strength and performance.

	Total sample	SARC-F		p	GRIP STRENGTH		p	SPPB		p
	(N = 259)	≥ 4 (N = 32)	< 4 (N =222)		Reduced * (N = 121)	Normal (N = 134)		≤ 8 (N = 14)	> 8 (N = 238)	
Weight (kg) 1 month after hospital discharge	77 (IQR 66 - 87)	69.5 (IQR 59.2 - 77.7)	78.0 (IQR 67 - 88)	**0.004**	73 (IQR 62 - 84)	80.0 (IQR 70.0 - 88.0)	**< 0.001**	67 (IQR 59 - 77.25)	77.0 (IQR 67.0 - 88.0)	**0.02**
BMI (kg/m2) 1 month hospital discharge	27 (IQR 24.22 - 29.47)	26.75 (IQR 23.10 – 30.27)	27 (IQR 24.34 - 29.41)	0.57	26.6 (IQR 23.42 - 29.34)	27.1 (IQR 24.74 - 29.71)	0.14	26.7 (IQR 22.10 - 28.39)	27.1 (IQR 24.43 - 29.69)	0.22
Chronic therapies 1 month after hospital discharge	3 (IQR 1 - 6)	6 (IQR 4 - 11)	3.0 (IQR 1.0 - 6.0)	**< 0.001**	5 (IQR 3 – 7)	2.0 (IQR 1.0 - 5.0)	**< 0.001**	8 (IQR 3.75 - 12.25)	3.0 (IQR 1.0 - 6.0)	**0.002**
Muscle thickness (cm) 1 month after hospital discharge	1.7 (IQR 1.44 - 1.93)	1.6 (IQR 1.33 - 1.80)	1.7 (IQR 1.45 – 1.95)	0.17	1.6 (IQR 1.36 - 1.87)	1.73 (IQR 1.52 – 1.99)	**0.02**	1.3 (IQR 1.14 - 1.65)	1.71 (IQR 1.47 – 1.96)	**0.01**
Pennation angle (°) 1 month after hospital discharge	22.4 (IQR 19.70 - 26.0)	22 (IQR 18.0 - 24.8)	22.9 (IQR 19.80 - 26.20)	0.20	22 (IQR 19.12 - 26.0)	23.0 (IQR 20.37 - 26.55)	0.15	20.0 (IQR 16.75 - 23.90)	22.8 (IQR 19.85 - 26.10)	0.06
Muscle stiffness 1 month after hospital discharge**	81.43 (IQR 65.02 – 97.32)	103.0 (IQR 94.73 - 111.0)	79.55 (IQR 62.57 – 93.98)	**< 0.001**	87.0 (IQR 74.82 – 103.10)	76.3 (SD ± 22.72)	**0.004**	104.9 (IQR 82.81 - 110)	81.07 (IQR 64.03 – 97.23)	**0.04**
MNA-SF 1 month after hospital discharge	9 (IQR 8 - 11)	8 (IQR 7.0 - 10.0)	9 (IQR 8 - 11)	**0.007**	9.0 (IQR 7 – 11)	9.0 (IQR 8.0 - 11.0)	0.08	8.5 (IQR 6 - 10)	9.0 (IQR 8.0 - 11.0)	0.07
SPPB 1 month after hospital discharge	12 (IQR 12 - 12)	11 (IQR 8.0 - 12.0)	12 (IQR 12 - 12)	**< 0.001**	12 (IQR 10.25 - 12)	12.0 (IQR 12.0 - 12.0)	**< 0.001**	7.5 (IQR 5 - 8)	12.0 (IQR 12.0 - 12.0)	**< 0.001**
Hand Grip Strength (kg) 1 month after hospital discharge	21.7 (IQR 15.95 – 30.0)	14.5 (IQR 11.35 - 16.1)	24.3 (IQR 17.5 – 31.3)	**< 0.001**	15.9 (IQR 12.8 - 22.25)	29.25 (IQR 19.63 – 33.97)	**< 0.001**	12.2 (IQR 10.95 - 15.90)	23.2 (IQR 16.72 - 30.70)	**< 0.001**
SARCF 1 month after hospital discharge	1 (IQR 0 - 2)	4.5 (IQR 4.0 - 5.0)	1 (IQR 0 - 2)	**< 0.001**	2 (IQR 1 - 3)	0.0 (IQR 0.0 - 1.0)	**< 0.001**	4 (IQR 2.75 - 5)	1.0 (IQR 0.0 - 2.0)	**< 0.001**

*Hand grip strength < 27 kg in men; < 16 kg in woman.

**data on muscle stiffness were available for only 152 patients.

SARC-F, Screening tool for sarcopenia; SPPB, Short Physical Performance Battery; IQR, Inter Quartile Range; BMI, Body Mass Index; MNA-SF, Mini Nutritional Assessment Short Form. Bold = statistically significant (p < 0.05).

**Figure 1:**
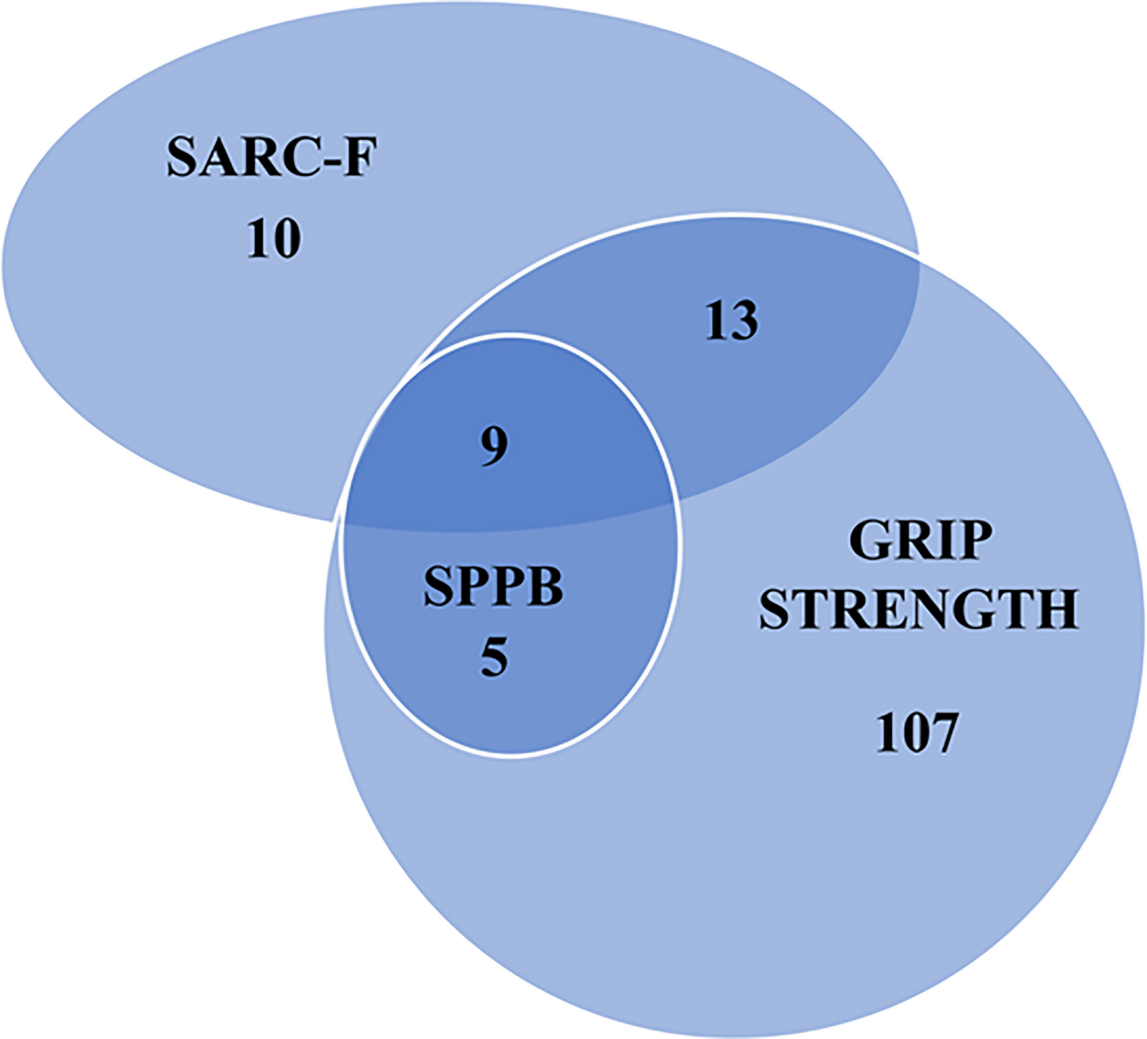
Number of people with a pathologic value of a sarcopenia screening tool, hand grip strength and muscle performance test. SARC-F, Screening tool for sarcopenia; SPPB, Short Physical Performance Battery.

The comparisons between patients with pathological *versus* normal values of muscle function and of SARC-F are shown in **Tables 1**–**3**. Many patients presented an overlap of pathological tests as illustrated by the Venn Diagram in **Figure 1**.

**Figure 2:**
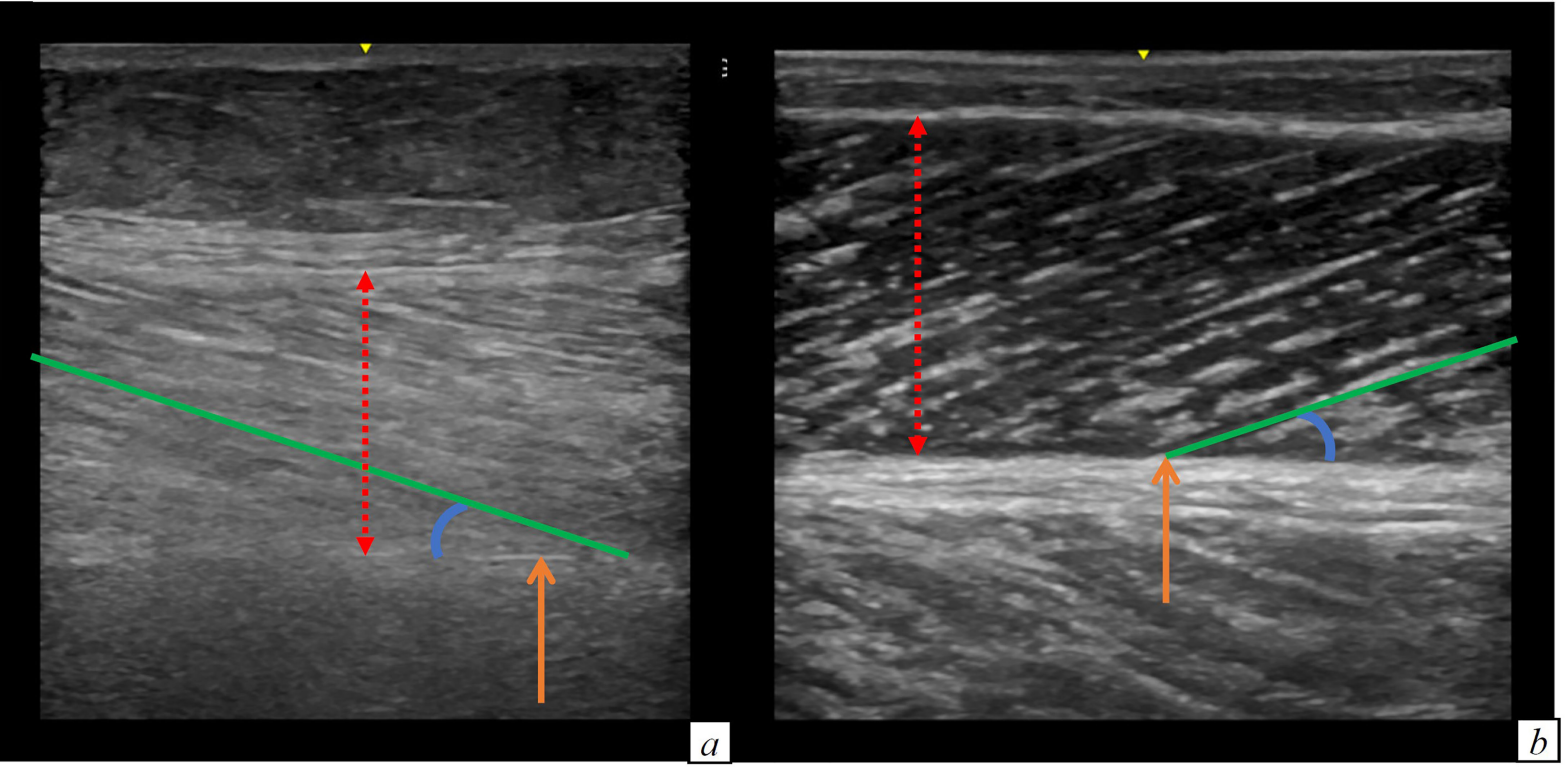
Comparison of the limb ultrasound images of a patient with **(A)** and without probable sarcopenia **(B)**. **(A)** Muscle thickness: 1.54 cm, muscle stiffness 127 **(B)** Muscle thickness: 1.81 cm, muscle stiffness 46.8. RED DOTTED ARROW: muscle thickness; ORANGE ARROW: muscle deep aponeurosis; BLU CIRCLE LINE: pennation angle; GREEN line: muscle fascicle length.

The patients with an impaired muscle function or with a pathological SARC-F score were older and more often females, compared to individuals with a normal muscle function. They had a lower weight and a higher burden of chronic therapies, both before hospital admission and one month after hospital discharge. Furthermore, their muscle stiffness was higher. Pennation angle and weight loss during hospital stay did not differ. Muscle thickness was significantly lower in patients with reduced versus those with normal muscle strength (1.6 versus 1.73 cm, p = 0.02). This finding was confirmed in patients with reduced versus those with normal muscle performance (1.3 versus 1.71 cm, p = 0.01). Muscle stiffness was higher in patients with reduced muscle strength compared to patients with normal muscle strength (87 versus 76.3, p = 0.004). Also in patients with reduced muscle performance muscle stiffness was higher compared to patients with normal muscle performance (104.9 versus 81.07, p = 0.04). **Figure 2** illustrates the muscle US images of a patient with reduced muscle strength (i.e. probable sarcopenia) and of a patient with normal muscle strength.

Osteoporosis (p < 0.001), dementia (p = 0.005) and vitamin D deficiency (p = 0.008) were more prevalent in people with a high SARC-F score, as compared to those with normal values. Moreover, the patients with a high SARC-F score had higher serum ferritin during hospital stay (p = 0.03), and worse nutritional status, one month after hospital discharge (MNA-SF: 8 versus 9, p = 0.007).

The patients with probable sarcopenia were more frequently affected by hypertension (p= 0.04), diabetes (p= 0.008), stroke or previous transient ischemic attack (p= 0.047), chronic obstructive pulmonary disease (COPD) (p = 0.04), neurological diseases, different from dementia (p = 0.01) and vitamin D deficiency (p = 0.03). Moreover, admission to the Intensive Care Unit was more frequent (p= 0.04), and WBC (p = 0.01) and days of C Reactive Protein (CRP) above the upper normal limit during hospitalization (p < 0.001) were higher in patients with probable sarcopenia.

The patients with a pathological muscle performance were more comorbid than patients with normal muscle performance. In particular, they were more frequently affected by hypertension (p= 0.03), ischemic heart disease (p=0.03), arrhythmia (p = 0.003), COPD (p= 0.01), chronic anaemia (p = 0.03), dementia (p = 0.006) and other neurological (p = 0.002) and psychiatric diseases (p = 0.048). In addition, during hospitalization, they had CRP levels above the upper normal limit, for a longer time (p = 0.03).

**Table 4** shows the results of the Spearman correlations among muscle ultrasound characteristics, and the measures of muscle function and nutritional status, age, inflammatory indexes, and length of hospital stay.

**Table 4:** Spearman correlations among muscle ultrasound characteristics, measures of muscle function and nutritional status, age, inflammatory indexes and length of hospital stay.

		Muscle thickness	Pennation angle	Muscle stiffness	MNA-SF	SPPB	Grip Strength	SARCF	Hospitalization length	Highest CRP	Days of elevated CRP	Highest ferritin	Highest WBC	Age
Muscle thickness	p		**0.46**	-0.05	**0.16**	**0.2**	**0.32**	**-0.23**	-0.02	0.05	-0.05	**0.16**	0.02	**-0.35**
	p		**< 0.001**	0.54	**0.01**	**0.001**	**< 0.001**	**< 0.001**	0.73	0.4	0.44	**0.01**	0.71	**< 0.001**
Pennation angle	p	**0.46**		-0.11	**0.17**	**0.13**	**0.16**	**-0.19**	-0.11	-0.01	-0.09	0.06	-0.004	**-0.21**
	p	**< 0.001**		0.17	**0.008**	**0.03**	**0.01**	**0.005**	0.07	0.9	0.15	0.33	0.95	**0.001**
Muscle stiffness	p	-0.05	-0.11		**-0.26**	-0.11	**-0.26**	**0.3**	0.15	0.04	0.13	-0.13	0.003	**0.22**
	p	0.54	0.17		**0.002**	0.19	**0.001**	**< 0.001**	0.07	0.66	0.10	0.12	0.97	**0.007**

MNA-SF, Mini Nutritional Assessment-Short Form; SPPB, Short Physical Performance Battery; SARC-F, Screening tool for sarcopenia; WBC, White Blood Cells. Bold = statistically significant (p < 0.05).

We detected significant direct correlations, between muscle thickness and pennation angle (p 0.46, p < 0.001), MNA-SF (p 0.16, p = 0.01), grip strength (p 0.32, p < 0.001), SPPB (p 0.2, p = 0.001) and ferritin (p 0.16, p = 0.01) and an inverse correlation of muscle thickness with SARC-F (p -0.23, p < 0.001) and with age (p -0.35, p < 0.001).

Pennation angle had a direct correlation with nutritional status (p 0.17, p = 0.008), and with muscle function (Hand Grip Strength: p 0.16, p = 0.01; SPPB: p 0.13, p = 0.03) and an inverse correlation with SARC-F (p - 0.19, p = 0.005) and with age (p -0.21, p = 0.001). Instead, muscle stiffness showed a direct correlation with age (p 0.22, p = 0.007) and with SARC-F (p 0.3, p < 0.001). Muscle stiffness had an inverse correlation with grip strength (p -0.26, p = 0.001), and with MNA-SF (p -0.26, p = 0.002).

At the univariable binary logistic regression model age (OR 1.07, 95% C.I. 1.04 – 1.09, p < 0.001), the number of chronic therapies at hospital admission (OR 1.24, 95% C.I. 1.13 – 1.35, p < 0.001), length of hospital stay (OR 1.07, 95% C.I. 1.04 – 1.1, p < 0.001), ICU stay (2.61, 95% C.I. 1.03 – 6.63, p = 0.04), NIV use during hospital stay (OR 2.09, 95% C.I. 1.18 – 3.70, p = 0.01), days of CRP above the upper normal limit (OR 1.08, 95% C.I. 1.04 – 1.11, p < 0.001) and muscle stiffness (OR 1.02, 95% C.I. 1.01 – 1.04, p = 0.003) resulted significant predictors of probable sarcopenia. Muscle stiffness (adjusted OR 1.02, 95% C.I. 1.002 – 1.04, p = 0.03) confirmed to be a significant predictor of probable sarcopenia, in the stepwise multivariable model adjusted for age, sex, NIV, and number of chronic therapies.

Cut-offs for probable sarcopenia identified by the ROC analyses were as follows: 1.51 cm for muscle thickness (AUC 0.59, 95% C.I. 0.52 - 0.66 p = 0.017, sensitivity 41%, specificity 76%) and of 73.95 for muscle stiffness (AUC 0.64, 95% C.I. 0.55 – 0.73, p = 0.004, sensitivity 77%, specificity 48%). The results for pennation angle were not statistically significant (AUC 0.55, 95% C.I. 0.48 – 0.62, p = 0.15). ROC curves are illustrated in **Figures 1S–3S**.

## Discussion

In this observational study, we found that one month after hospital discharge, COVID-19 survivors with reduced muscle function displayed low muscle mass and increased muscle stiffness at the ultrasound evaluation of the dominant medial gastrocnemius as compared with those with normal muscle function. The finding of increased muscle stiffness was confirmed in patients with a pathological SARC-F score too. Moreover, we detected a significant correlation between muscle ultrasound parameters and age, nutritional status and muscle performance. Finally, we detected an association between muscle stiffness and probable sarcopenia and with the ROC analyses we identified the cut-offs of the muscle ultrasound parameters, associated with probable sarcopenia.

Our findings refer to preliminary data, collected one month after hospital discharge. The 3 and 6 months follow ups of the patients of this study are ongoing. Indeed, it is of paramount importance to continue follow up visits over time, because it has been reported that musculoskeletal symptoms can persist 3 and 6 months after hospitalization in COVID-19 survivors (45). Assessing whether these symptoms are underpinned by alteration of muscle function, mass and quality will allow the characterization of the COVID-19 disease on muscles, and the long-term effects of acute sarcopenia, that are presently unknown (46).

Our results on the negative correlation between both muscle thickness and age, and pennation angle and age are in line with the typical architectural remodelling of ageing (47) characterized by decreased muscle size, and reduced pennation angles (48). Indeed, muscle thickness and pennation angle are characteristics of the muscle architecture, that are strongly related one to the other, as previously demonstrated by Kubo (49), and as confirmed in our study.

Detecting changes in muscle architecture is of extreme importance, since these alterations have an impact on the mechanical behaviour of muscles (50). Our study confirmed the presence of a significant correlation between muscle ultrasound characteristics and measures of muscle function. Moreover, we found a significant correlation between muscle ultrasound aspects and nutritional status evaluated with the MNA-SF. Malnutrition, particularly when disease-associated, is known to be associated with alterations in body composition and reductions in fat free mass (51). Both the European Society of Clinical Nutrition and Metabolism (52) and the Global Leadership Initiative on Malnutrition (53) guidelines recommend the evaluation of fat free mass as a diagnostic criterion for malnutrition. Therefore, the association of a malnutrition screening tool with impaired measures of muscle mass and quality is not surprising. Muscle ultrasound is a simple and non-expensive tool to assess skeletal muscle characteristics, and could be a valuable instrument for the screening of malnutrition. Our finding is in line with the study by Mateos-Angulo et al. (54) that detected an association between MNA-SF and muscle thickness, measured with ultrasonography, in istitutionalized older adults.

Compared to the study of Minetto et al. (55), the median values of muscle thickness were higher in our population (1.7, IQR 1.44 - 1.93 cm in the total sample of our study versus 1.42 ± 0.03 cm in men and 1.23 ± 0. 28 cm in women in the study of Minetto). However, Minetto et al. considered a small sample (44 people) of institutionalized pre-frail and frail older adults (mean age 79.2 ± 8.3 years) whereas our study included 259 community dwelling people with a median age of 67 years (IQR 56 – 75). Our data on muscle thickness are more in line with the findings of Kubo et al. (49) who detected a mean muscle thickness of 1.93 ± 0.27 cm in community dwelling men (mean age 69.5 ± 4.2 years) and of 1.77 ± 0.23 cm in community dwelling women (mean age 68.0 ± 5.3 years).

Differently from Kubo, the median values of the pennation angle, were higher in our sample (22.4° versus 16.5° in men and 15.6° in women). Anyway, the measurement of the pennation angle is strongly influenced by the pressure that the sonographer exerts on the muscle, and further data are needed to define normal and pathological values of this parameter in older people.

Our study showed that muscle stiffness is augmented in post COVID-19 patients with reduced muscle function and pathological SARC-F score, as compared with those who had normal values of muscle function and SARC-F. Since we measured muscle stiffness with muscles in a resting condition, our finding refers to passive muscle stiffness. Passive muscle stiffness is an important characteristic, since it regulates the interactions between body and environment. When muscle stiffness is too elevated, the energy of the body-environment interactions can be transmitted to the tissues, causing an injury (56). For example, in people with an elevated muscle stiffness, there is a higher risk of muscle damage after eccentric exercise (57, 58).

Passive muscle stiffness is influenced by collagen deposition, inflammation and swelling (59–61). Previous studies showed that the amount of collagen, of advanced glycation end-products and of collagen cross-linking in connective tissue, increase with ageing (62, 63). Indeed, we found a significant correlation between muscle stiffness and age. In addition to increasing muscle stiffness [as demonstrated in aged (64) and sarcopenic muscles (65)], the alterations of the extracellular matrix may also favour muscle mass decrease. The alterations in muscle extracellular matrix can alter the regenerative potential of the myogenic progenitor cells (66). However, the exact relation between muscle stiffness and aging has not been clearly elucidated so far. While some studies demonstrated higher muscle stiffness in older people (67–70), others detected opposite results (71). Our findings are in line with the first ones.

In this study we identified possible muscle ultrasound parameters cut-offs, for probable sarcopenia. Muscle ultrasound is a non-invasive, little expensive and low time-consuming technique. As such, it could potentially be considered an optimal screening test for probable sarcopenia. In our study, the muscle thickness cut off for probable sarcopenia had a high specificity (76%), but a low sensitivity (41%). It is known that highly specific screening tests unlikely yield false positive results (72). Therefore, people with a pathologic muscle thickness would likely have probable sarcopenia. On the contrary, the ultrasound cut-off for muscle stiffness had a high sensitivity (77%) but a low specificity (48%). Highly sensitive screening tests unlikely generate false negative outcomes (72). Thus, people with a normal muscle stiffness would not have probable sarcopenia.

Unfortunately, the AUC of the ROC curves were < 0.7. These results indicate that muscle ultrasound has a low accuracy in detecting probable sarcopenia, compared to the gold standard hand grip test. Anyway, these results refer to a preliminary and reduced sample, and could be improved by future wider studies. Moreover, muscle ultrasound could be used as a complementary technique to hand grip test to assess the morphologic characteristics of skeletal muscle in patients with probable sarcopenia.

Our study has the merit of having described for the first-time muscle mass and characteristics of post COVID-19 patients with the use of limb muscle ultrasound. Description of muscle ultrasound parameters of post COVID-19 patients with impaired muscle function and pathological SARC-F score is important, since no accepted definition of muscle quality exists so far. Characterizing the changes of muscle architecture through a non-invasive and easy to use tool as echography would provide information to better define muscle quality. Finally, we identified possible cut-off values of the muscle ultrasound parameters suggestive of the risk of sarcopenia in post COVID-19 patients. It could be speculated that muscle ultrasonography may detect subjects slowly recovering from COVID-19, and with potentially negative long-term sequelae.

Some limitations of this study deserve to be mentioned: the relatively limited sample size, the fact that some patients with dementia (in the absence of their care-givers) could have improperly answered to some questions of the SARC-F, the missing information on muscle stiffness for 107 patients, and the dependency on the ability of the operator for the evaluation of muscle mass and quality. Due to the lack of measures of muscle mass/function prior to SARS-CoV-2 infection or during hospitalization, we could not specifically address the impact of COVID-19 on skeletal muscle. However, the main aim of our study was to characterize muscle mass and quality by muscle ultrasound in a population prone to skeletal muscle impairment (19, 20), and to assess the association of ultrasound parameters with established tools for the assessment of the risk of sarcopenia. Further studies are needed to assess whether our findings can be generalized to patient populations other than COVID-19 survivors. Finally, an important limit is that we did not compare ultrasound muscle characteristics against reference methods for measuring fat free mass, such as dual-energy X-ray absorptiometry, CT or MRI. In the future, wider, multicenter studies will help better define the role of ultrasound for the evaluation of muscle quantity and quality, and correlate these data to relevant clinical outcomes.

In conclusion, we showed that muscle ultrasound parameters have a significant correlation with age, nutritional status and muscle performance in COVID-19 survivors. Although our findings need to be confirmed by studies comparing muscle ultrasound against validated techniques for measuring muscle mass and quality, our study suggests, for the first time, that muscle ultrasound could be an innovative tool to assess muscle mass and quality in COVID-19 survivors.

## Data Availability Statement

The raw data supporting the conclusions of this article will be made available by the authors, without undue reservation.

## Ethics Statement

The studies involving human participants were reviewed and approved by San Raffaele University Hospital Ethics Committee (protocol no. 34/int/2020). The patients/participants provided their written informed consent to participate in this study.

## Author Contributions

All authors made substantial contributions to all of the following: (1) the conception and design of the study, or acquisition of data, or analysis and interpretation of data, (2) drafting the article or revising it critically for important intellectual content, (3) final approval of the version to be submitted.

## Funding

This study was financially supported by Ministero della Salute, Italy, and by COVID-19 donations.

## Conflict of Interest

The authors declare that the research was conducted in the absence of any commercial or financial relationships that could be construed as a potential conflict of interest.

## Publisher’s Note

All claims expressed in this article are solely those of the authors and do not necessarily represent those of their affiliated organizations, or those of the publisher, the editors and the reviewers. Any product that may be evaluated in this article, or claim that may be made by its manufacturer, is not guaranteed or endorsed by the publisher.
