# Supplementary material for: Evaluation of Muscle Mass and Stiffness with Limb Ultrasound in COVID-19 Survivors
Source: Front Endocrinol (Lausanne). 2022 Feb 17;13:801133. doi: 10.3389/fendo.2022.801133 (PMC8892603; doi:10.3389/fendo.2022.801133)
Supplement: Supplementary file 1 [file DataSheet_1.pdf]

Figure 1S: ROC curve for the values of muscle thickness associated with probable sarcopenia

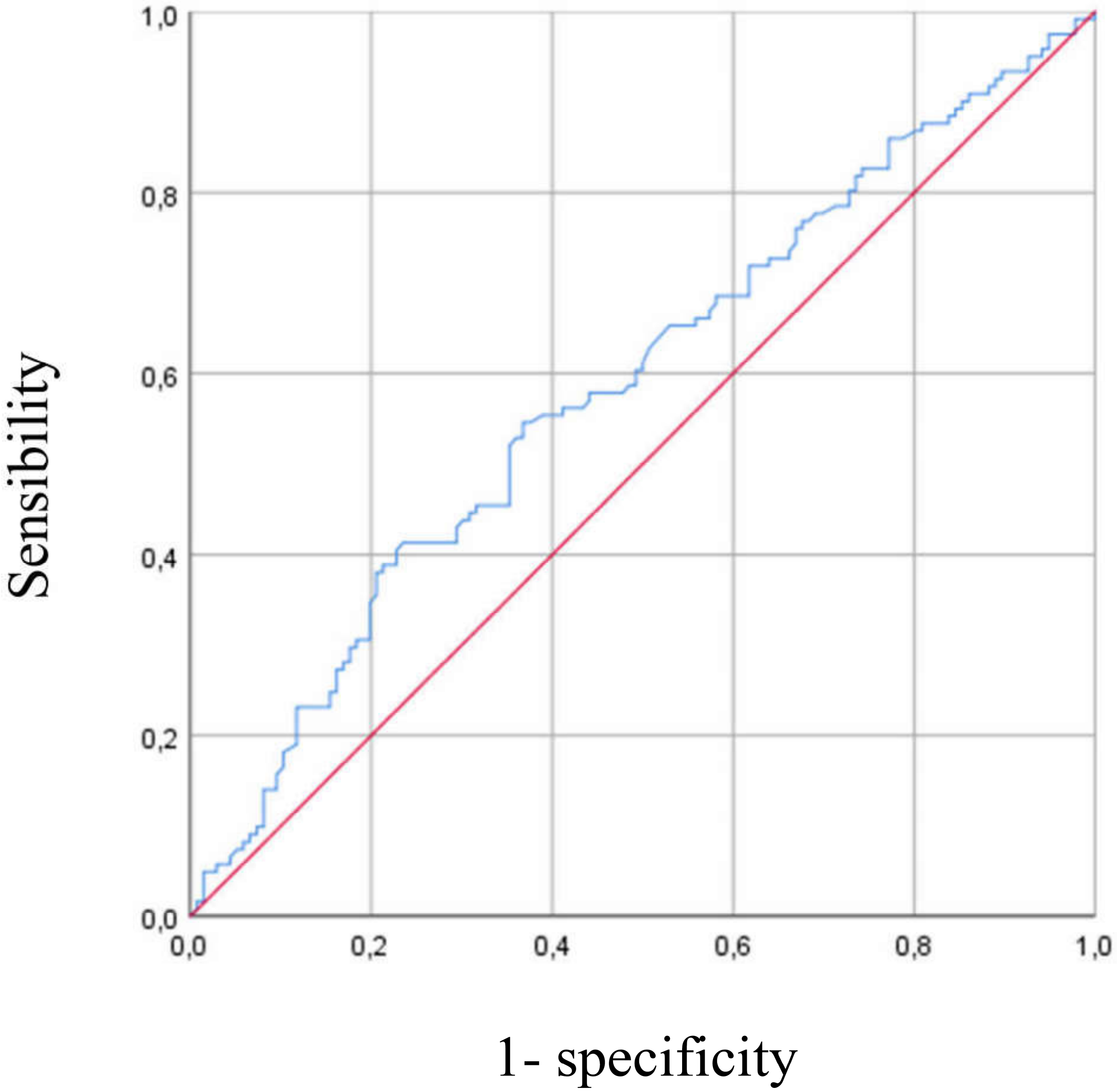

State variable: Grip strength, AUC = 0.59, p = 0.017, cut off = 1.51 cm, sensibility = 41%, specificity 76%

Figure 2S: ROC curve for the values of pennation angle associated with probable sarcopenia

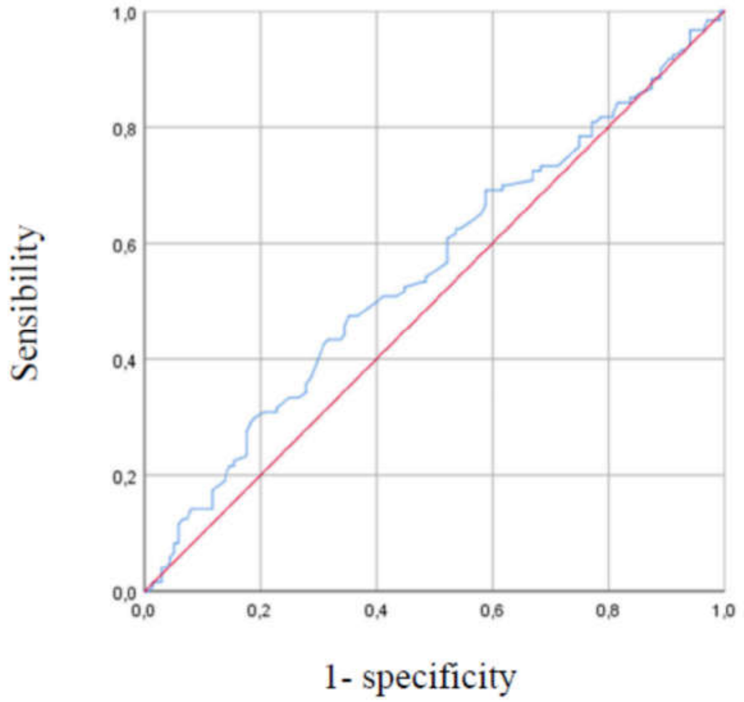

State variable: Grip strength, AUC = 0.55, p = 0.15, cut off = n.a., sensibility =n.a., specificity n.a.

n.a. = not applicable

Figure 3S: ROC curve for the values of muscle stiffness associated with probable sarcopenia

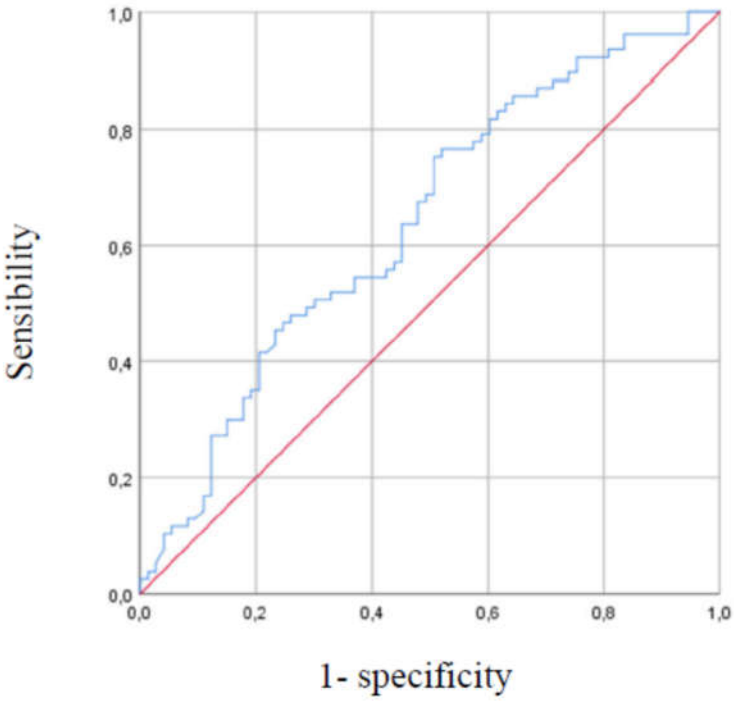

State variable: Grip strength, AUC = 0.64, p = 0.004, cut off = 73.95, sensibility = 77%, specificity 48%
